# Retrograde flow phenomenon of poly‐D,L‐lactic acid filler injection over lower eyelids

**DOI:** 10.1002/ski2.377

**Published:** 2024-03-21

**Authors:** Chuan‐Yuan Lin, Jui‐Yu Lin

**Affiliations:** ^1^ Li‐An Medical Clinic Taipei City Taiwan

## Abstract

The occurrence of ‘retrograde flow along the cannula/needle tract’ during filler injections has not been extensively addressed in the existing literature, possibly because it is often imperceptible unless the injection is conducted at a superficial layer. In our experience with poly‐D,L‐lactic acid filler for lower eyelid injections, we have observed and documented this phenomenon through the skin. This article aims to elucidate and illustrate the retrograde flow phenomenon and discuss the factors influencing it.

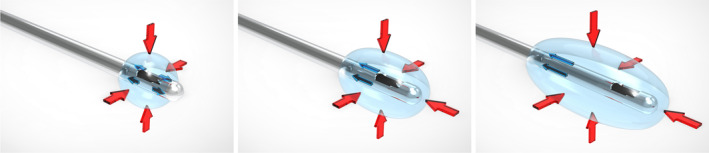

Dear Editor, The occurrence of ‘retrograde flow along the cannula/needle tract’ during filler injections has not been extensively addressed in the existing literature,^1^, ^2^ possibly because it is often imperceptible unless the injection is conducted at a superficial layer. In our experience with poly‐D,L‐lactic acid (PDLLA; AestheFill; REGEN) filler for lower eyelid injections,^3^, ^4^ we have observed and documented this phenomenon through the skin. This article aims to elucidate and illustrate the retrograde flow phenomenon and discuss the factors influencing it.

PDLLA proves effective for lower eyelid rejuvenation through a method involving a superficial, wide, and even distribution of PDLLA under the eyes.^3^ To achieve even distribution, the ‘Fan‐dotting technique’ was introduced by Lin et al.^4^ In this technique, a 23G cannula is inserted beneath the dermis, starting 1 cm away from and below the outer corner of the eye. The injection involves administering a small amount of PDLLA, retracting the cannula slightly, and repeating these steps until the desired area is adequately injected. Each dot's administered amount typically ranges from 0.02 to 0.033 mL, with a 3–4 mm gap between consecutive dots.^4^ This method allows the observation of the ‘retrograde flow’ phenomenon through the eyelid skin after injecting a single dot of filler. In our experience, this retrograde flow contributes to a more uniform filler distribution during the injection process (Video 1).

The retrograde flow phenomenon can be described as follows: as the cannula creates a tract in the subdermal tissue, PDLLA filler can spread within this potential space. This is because filler material tends to spread towards areas of least resistance.^1^ The injection process involves pushing the surrounding tissue away from the cannula tip opening, and the rebounding forces of the tissue compress the filler, causing it to extend both distally and proximally along the tract. The proximal extension, longer and more visible, results in the termed ‘retrograde flow’ (Figure 1).

**FIGURE 1:**
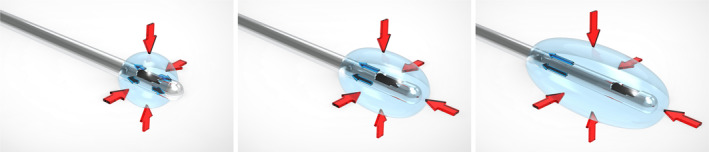
Schematic diagrams illustrate the mechanism of retrograde flow of PDLLA filler in the sub‐dermal plane using cannula injection. (Left) When PDLLA injection begins, it displaces the surrounding tissue away from the cannula, leading to the generation of rebounding forces within the tissue that compress the filler. (Middle) With continued injection, the filler extends both distally and proximally along the cannula tract. (Right) However, the filler predominantly extends proximally along the cannula tract, as the distal cannula tract is relatively short. PDLLA: poly‐D,L‐lactic acid; Red arrows: rebounding forces generated by the tissue; Blue arrows: directions of filler extension.

According to rheology, the extent of flow depends on the filler's cohesivity and the applied compression force.^5^ Cohesivity, representing the behaviour of the filler as it interacts within the tissue once it has been implanted, is the internal adhesive force that bind together the individual filler units in a deposit.^5^ Consequently, when injecting different fillers into lower eyelids (i.e., with the same compression force), a filler with lower cohesivity will create a longer retrograde flow. This explains, in few of our practical experiences, accidental air injection into the subdermal layer due to neglecting to expel air from the cannula before injecting filler results in a filling of the cannula tract with air. On the other hand, injecting filler into tissues with higher elasticity (i.e., with higher compression force) also leads to a longer retrograde flow. This is the reason why injecting a filler into scar tissue often results in a noticeable retrograde flow.^2^

Despite the crucial role of retrograde flow in achieving even filler distribution in superficial layer PDLLA injections for lower eyelid rejuvenation, further studies are needed on other influencing factors.^5^ First, PDLLA filler contains water and carboxymethylcellulose,^3^, ^4^ with water having lower cohesivity than carboxymethylcellulose. Theoretically, water can spread further than carboxymethylcellulose. Consequently, injecting a large amount of PDLLA filler in a single location may not be advisable.^4^ Additionally, filler spreading is also influenced by shearing force, which is correlated with injection speed.^5^ Other factors such as needle/cannula size and the volume of filler injected may also affect retrograde flow. However, these factors have a relatively low impact due to the typical slow, steady injection pattern over lower eyelid regions.

In conclusion, the retrograde phenomenon is a unidirectional lateral dispersion of filler material along cannula/needle tract where is the area of least resistance. The primary factors influencing this phenomenon are filler cohesivity and compression force applied during injection. This article has presented the retrograde flow of PDLLA in the sub‐dermal layer of lower eyelid. Practitioners can leverage this property for even distribution of a low cohesivity PDLLA filler over superficial layer of lower eyelid regions using the fan‐dotting method.

## CONFLICT OF INTEREST STATEMENT

Dr. Lin J‐Y and Dr. Lin C‐Y are medical consultants of Jiangsu Wuzhong Aesthetic Biotech., and medical directors of REGEN Biotech.

## AUTHOR CONTRIBUTIONS

**Chuan‐Yuan Lin**: Conceptualization (lead); data curation (lead); formal analysis (lead); writing – original draft (lead); writing – review & editing (lead). **Jui‐Yu Lin**: Conceptualization (supporting); data curation (supporting); formal analysis (supporting); writing – original draft (supporting); writing – review & editing (supporting).

## FUNDING INFORMATION

This research received no specific grant from any funding agency in the public, commercial, or not‐for‐profit sectors.

## ETHICS STATEMENT

Patients provided written consent for the use of their images.

## Supporting information

Supporting Information S1

Supporting Information S2

## Data Availability

Data sharing not applicable to this article as no datasets were generated or analysed during the current study.
