# Supplementary material for: Retrograde flow phenomenon of poly‐D,L‐lactic acid filler injection over lower eyelids
Source: Skin Health Dis. 2024 Mar 21;4(3):e377. doi: 10.1002/ski2.377 (PMC11150744; doi:10.1002/ski2.377)
Supplement: Supplementary file 1 — Supporting Information S1 [file SKI2-4-e377-s002.docx]

**Caption of Video 1**

Video 1: An example of retrograde flow phenomenon by poly-D,L-lactic acid filler injection over lower eyelid.
